# Myocardial Energetics in Left Ventricular Hypertrophy

**DOI:** 10.2174/157340309788970379

**Published:** 2009-08

**Authors:** Mohammad N Jameel, Jianyi Zhang

**Affiliations:** Department of Cardiology, University of Minnesota, Minneapolis, MN, USA

**Keywords:** Left ventricular hypertrophy, myocardial energetic.

## Abstract

The heart carries out its pumping function by converting the chemical energy stored in fatty acids and glucose into the mechanical energy of actin-myosin interaction of myofibrils. Development of congestive heart failure is usually preceded by a period of compensated left ventricular hypertrophy (LVH) and alterations in myocardial bioenergetics have been considered to play an important role in this transition. Myocardial energetic state that is reflected by the ratio of Phosphocreatine to Adenosine Triphosphate (PCr/ATP) is significantly decreased in hearts with LVH. The severity of this abnormality is linearly related to the severity of cardiac hypertrophy as well as left ventricular (LV) dysfunction, and is independent of a persistent myocardial ischemia. The decrease in PCr/ATP is accompanied by a decrease in creatine kinase flux and alterations in substrate utilization in LVH hearts. Moreover, there is a profound heterogeneity in alterations in myocardial energy metabolism in hearts with post-infarction hypertrophy with the most severe abnormality present in the inner layers of the periscar border zone (BZ). This review will discuss various aspects of myocardial energetics in animal models of three different types of LVH (pressure-overload, volume overload and post-infarction) with a brief description of myocardial energetics in humans with LVH.

## INTRODUCTION

Cardiac energy metabolism has three components. The first is substrate utilization which involves the cellular uptake of substrates (mainly fatty acids and glucose) and their breakdown by beta-oxidation and glycolysis resulting in the formation of acetyl coenzyme A (CoA), which is fed into the Krebs cycle and produces Nicotinamide Adenine Dinucleotide (NADH) and carbon dioxide (CO_2_). The second component is oxidative phosphorylation which involves transfer of electrons from NADH to oxygen through the mitochondrial respiratory chain that creates a proton electrochemical gradient across the inner mitochondrial membrane subsequently driving the F1-F0 ATP synthase to produce Adenosine Triphosphate (ATP) by phosphorylating Adenine Diphosphate (ADP). The third component is ATP transfer and utilization that involves the creatine kinase shuttle. 

Derangements in cardiac energy metabolism occur with the development of left ventricular hypertrophy (LVH). LVH is a manifestation of cardiac remodeling and has been classified into pressure overload hypertrophy, volume overload hypertrophy and post-infarction hypertrophy. LVH is initially adaptive but ultimately leads to maladaptive hypertrophy and transition to congestive heart failure (CHF). The exact mechanisms underlying this transition from compensated LVH to CHF are likely multifactorial but alterations in myocardial bioenergetics have been considered to play an important role.

The principal method for studying myocardial energy metabolism *in vivo *is through Nuclear Magnetic Resonance (NMR) spectroscopy. Our laboratory has specialized in performing *in vivo *NMR spectroscopy in large animals to study abnormalities in high energy phosphate metabolism, oxidative phosphorylation, creatine kinase kinetics and flux and substrate utilization. In this review, we will focus on the myocardial energetics in different animal models of ventricular hypertrophy (summarized in Table **1**).

## PRESSURE OVERLOAD HYPERTROPHY

### Animal Model

Pressure overload hypertrophy leads to concentric hypertrophy due to an increase in wall thickness that can be seen clinically in patients with hypertension or aortic stenosis. Different animal models have been used to create pressure overload leading to concentric hypertrophy. The degree of ventricular hypertrophy varies in these models and thus the results of the studies vary slightly. The most commonly used method is placement of a constrictor band around the ascending aorta to cause an increase of left ventricular systolic pressure. 

### High Energy Phosphate Levels

In our laboratory, we placed constrictor bands on the ascending aorta in young 8 week old dogs to create a peak systolic pressure gradient of approximately 20-30mmHg across the stenosis [1]. The pressure overload then increased progressively as the degree of aortic narrowing remained fixed in the face of normal body growth. After a follow-up of 1 year, LVH expressed as a ratio of left ventricular weight to body weight (LVW/BW) was significantly higher in the banded dogs as compared to normal controls (8.9±0.7 g/kg* vs. *4.3±0.14 g/kg, p< 0.01) which represented a doubling of the relative LV mass in the banded dogs [1]. None of the animals developed clinical evidence of heart failure. There was a significant decrease in high energy phosphates and PCr/ATP ratio in the LVH dogs under baseline conditions [1]. Specifically, ATP was decreased by 42%, Creatine Phosphate (CP) by 58%, and the CP/ATP ratio by 32% in comparison with normal dogs. There was a transmural gradient with PCr/ATP ratios of 1.62±0.15, 1.61±0.11 and 1.43±0.06 in the subepicardium, midwall and subendocardium respectively. These energetic abnormalities were proportional to the degree of hypertrophy. Moreover, ADP levels calculated from the creatine kinase equilibrium increased proportionally with the severity of LVH. These high energy phosphate (HEP) abnormalities were not corrected by pharmacological vasodilatation with adenosine, thus implying that these energetic changes are not due to persistent hypoperfusion [1]. When the hypertrophied heart was placed under stress with atrial pacing at 200 and 240 bpm, there was a further decrease in the creatine phosphate and accumulation of inorganic phosphate in the endocardial layers and this correlated with a decreased blood flow in these layers. These pacing induced abnormalities were not seen in normal hearts. 

Using a similar method of aortic banding in adult dogs, moderate LVH can be created [2]. In these dogs, a pressure gradient of about 50mmHg was created across the narrowing and the dogs were followed for about 2 months. This resulted in a LVW/BW of 5.7±0.19g/kg in the banded dogs which was 32% greater than normal animals [2]. In these dogs, the basal HEP levels and PCr/ATP ratio was normal as compared to the abnormal values in the severe LVH model mentioned above. However, pacing at 240 bpm resulted in similar decline in PCr and accumulation of inorganic phosphate (Pi) in the subendocardial layers. This was also associated with a relatively decreased blood flow to the endocardial layers. Dobutamine had a positive chronotropic and inotropic effect leading to greater increases in rate pressure product (RPP) in hypertrophied hearts as compared to normal which was associated with greater decreases in PCr/ATP that were uniform across the LV wall. When the dose of dobutamine was increased in normal animals to increase the RPP to similar amounts as the LVH animals, similar decreases in HEP levels was noted. Thus, dobutamine induced HEP abnormalities seem to be related to the increase in workload in these animals.

### Myocardial Oxygenation

Although, there was a relatively decreased blood flow to the endocardium with pacing in the above studies, it still does not imply that these tissues are ischemic. In another study, moderate LVH was created in young 2- 4 week old pigs with ascending aortic banding to create a systolic pressure gradient of 20mmHg [3]. This produced a 38% increase in LVW/BW after approximately 3 months. Coronary reserve, as assessed by the ratio of blood flow during adenosine infusion to baseline blood flow, was significantly lower in the LVH pigs compared with controls (3.5±0.4* vs. *5.5±0.4 mL/g · min, p<0.05); however, transmural myocardial blood flow was similar in both groups of pigs, both at baseline and with dobutamine stimulation, probably reflecting the higher coronary perfusion pressure in the LVH pigs. The PCr/ATP ratio tended to be lower in the LVH animals at baseline but both groups had a similar decline in the PCr/ATP ratio with dobutamine stimulation. Moreover, anterior wall lactate release did not differ between the groups at baseline, nor did the increase in lactate release differ during dobutamine stimulation. Thus, despite their reduced coronary vasodilator reserve and the absence of a greater rise in myocardial blood flow to compensate for a substantially higher LV double product, pigs with moderate LVH did not exhibit a greater susceptibility to myocardial ischemia during dobutamine stress [3]. 

It could still be argued that the HEP abnormalities seen in LVH hearts at baseline and higher work state are the result of impaired diffusion of oxygen to the cells. It has been observed that the intercapillary distances in hearts with pressure overload are increased. This is due to the fact that the capillary:myocyte ratio remains unchanged while the myocyte diameter increases. So, the oxygen diffusion distance is greater in hypertrophied hearts as compared to normal resulting in the possibility of oxygen deficiency at the mitochondria with no abnormality in the blood flow rates. H1 NMR spectroscopy has been used to quantitate the degree of myoglobin deoxygenation in myocytes and thus determine the intracellular oxygen tension. Using this technique, it has been demonstrated that the abnormally decreased PCr/ATP ratio found in severely hypertrophied myocardium during basal conditions cannot be ascribed to inadequate myocyte oxygen availability [4]. Secondly, the more prominent HEP changes during catecholamine stimulation in hypertrophied hearts are not associated with detectable deoxymyoglobin, thus failing to support impaired oxygen diffusion as a limiting factor in the hypertrophied myocardium [4].

### Myocardial Creatine Kinase Kinetics and Isoform Expression

The creatine kinase (CK) system plays an important role in myocardial energy metabolism by maintaining ADP levels high at the mitochondria, where ATP is generated and low at sites of ATP utilization [5]. Decreases of CK activity have the potential of affecting myocardial performance. In a canine model, ascending aortic banding in 8 week old dogs resulted in severe LV hypertrophy with LVW/BW of 9.45±0.63g/kg which was 92% greater than normal animals [6]. This resulted in significant decrease in myocardial PCr, creatine, and PCr/ATP as well as a 30% decrease in ATP. This was associated with a 40% decrease in CK-MM isoform mRNA and 50% decrease in CK-MM isoform protein and 20% decrease in CK-mitochondrial protein. On the other hand, CK-MB isoform mRNA was significantly increased and the CK-MB protein level was increased 10 fold as compared to normal animals. The CK-MB isoform increase in models of LVH cannot be merely attributed to a compensatory increase as a result of the decrease in CK-MM isoform as deletions of CK-MM and CK-mitochondria in isolated perfused mice heart models did not result in an increase in CK-MB protein [7]. Similarly, this fetal shift does not seem to offer any kinetic advantage as the CK velocity/CK Vmax *in vivo *was similar for CK-MB and CK-MM [7]. It could be a result of the hemodynamic overload on the LV or the consequent myocyte hypertrophy.

Total CK activity remained normal; however this does not take into account the CK compartmentalization within the cell. The higher CK-MB levels may contribute to total CK activity but CK-MB is not compartmentalized like CK-MM or CK-mitochondria within the cell. CK forward rate constant was not different in LVH animals but the CK forward flux rate was decreased by 60% in LVH and further decreased during dobutamine stimulation. The myocardial free ADP was increased in LVH animals. The increased ADP in association with a lower CK flux rate in the hypertrophied hearts mirrors similar responses to increases of workload that occur in the fetal or newborn heart and are consistent with the concept that an alteration in the pathway for mitochondrial regulation exists in the hypertrophied hearts that is similar to that in the less ordered fetal or neonatal heart. However, the CK flux rate was still 7-8 times higher than the ATP utilization rate and thus did not result in impairment of LV contractile function as shown by the increase in the LV systolic pressure and rate of ATP synthesis (MvO2) in response to dobutamine. Similar increases in CK-MB, decrease in the sum of CK-MM and CK-mitochondria with normal total CK activity have been found in a rat model of moderate LV hypertrophy (30% increase in LVW/BW) [8].

### Oxidative Capacity

Although myocardial HEP metabolism is abnormal in hearts with pressure overload hypertrophy and failure, it is unclear whether this is associated with impaired oxidative capacity. Mitochondrial uncoupling agent 2,4-dinitrophenol (DNP) has been used to determine whether the hypertrophied and failing hearts have reserve oxidative capacity during the high workload produced by catecholamine infusion [9]. This revealed that DNP increased MvO2 in swine with compensated hypertrophy but failed to do so in swine with overt heart failure. At the same time PCr/ATP was maintained in compensated hypertrophy while it decreased further in heart failure. Therefore, primary abnormalities of oxidative ATP production did not constrain contractile performance at moderately high workloads in hearts with compensated pressure overload hypertrophy, while the capacity of some rate limiting reaction in the oxidative ATP synthetic pathway may have been reached in swine with heart failure at high cardiac workstates.

### Substrate Utilization

Alterations of substrate metabolism have been reported in several models of LVH and failing hearts. Glucose and fatty acid (FA) oxidation was measured *ex vivo *in the spontaneously hypertensive rat (SHR) model of LVH [10]. These 15 week old rats had higher systolic blood pressures (189±8* vs. *139±7 mmHg) and LVH (HW/BW was 5.19±0.24* vs. *3.93±0.14 mg/g). The results revealed that FA oxidation of SHR hearts is profoundly suppressed (60-80%) relative to that of the normotensive Sprague Dawley (SD) strain, particularly at high workloads. Glucose oxidation is also moderately elevated, yielding a marked (four-to-five-fold) increase in the ratio of glucose/FA oxidation rates in the SHR hearts. Since more ATP is generated per mole of oxygen consumed when glucose is the fuel source, these results are consistent with the hypothesis that a shift away from FA use toward glucose contributes to the preservation of energetic economy in stable, concentric LVH. 

Accumulation of 2-deoxyglucose (2-DG) can be used as a surrogate of glucose uptake. Using intracoronary infusion of 2-DG, our group has shown that the severe pressure-overloaded hypertrophied myocardium accumulated ^31^P NMR–detectable 2DGP but normal myocardium did not, and that 2DGP accumulation in the hypertrophied hearts was greatest in the subendocardium [11]. Accumulation of 2DGP was related to the presence of myocardial hypertrophy and not to systolic pressure overload alone, since acute elevation of left ventricular systolic pressure in normal hearts did not result in detectable accumulation of 2DGP [11]. In another study of moderate LVH in pigs, the uptake of glucose, lactate, and free fatty acids did not differ between the groups in the basal state [3]. However, during dobutamine stimulation, glucose uptake was greater in the LVH group (0.84 +/- 0.09 mumol/g.min* vs. *0.59 +/- 0.08 mumol/g.min, P <.05). In a subset of animals, 14C-glucose was used to assess glucose oxidation. These data showed that the LVH animals had a greater rate of glucose oxidation (0.6 +/- 0.10* vs. *0.28 +/- 0.08 mumol/g.min, P <.05) and a greater rate of glucose conversion to lactate (0.20 +/- 0.04 versus 0.09 +/- 0.02 mumol/g.min, P < .05) compared with the control pigs. Thus, LVH pigs exhibited significantly greater use of exogenous glucose during dobutamine stress, as evidenced by increases in both glucose oxidation and anaerobic glycolysis. The increased myocardial free ADP levels seen in models of pressure overload hypertrophy can be partly explained by a change in substrate utilization in LVH animals. A decrease in fatty acid utilization and increase in glycolysis may result in lower NADH levels and lower NADH generation that would require a higher cytosolic ADP to drive ATP production to a level necessary to maintain the ATP utilization rate. 

It has also been shown that the reduction in fatty acid oxidation in hypertrophied hearts is accompanied by an increase in a compensatory metabolic pathway referred to as anaplerosis (filling up of the tricarboxylic acid (TCA) cycle) that provides an alternate route for glucose oxidation [12]. Transverse aortic constriction in rats resulted in 63% LVH after 10 weeks and 13C NMR spectroscopy was then used to study the oxidative metabolism in these isolated hearts that were perfused with palmitate and glucose. PCr/ATP ratio was decreased by 30% which was accompanied by a decrease in fatty acid oxidation. Flux through carnitine palmitoyltransferase I (CPT-I) was 23% lower in hypertrophied hearts at similar TCA cycle rates and was accompanied by a shift towards increased expression of the L–CPT I isoform (Liver isoform as compared to muscle isoform). Moreover, glucose oxidation via pyruvate dehydrogenase complex to produce acetyl CoA did not compensate for reduced palmitate oxidation rates. However, hypertrophied rats displayed an 83% increase in anaplerotic flux into the TCA cycle that was supported by conversion of glycolytic pyruvate into malate by malic enzyme whose levels were also increased. This increased oxidation of glucose through anaplerosis is inherently inefficient leading to decreased production of NADH.

Recent advances in molecular biology have enabled us to study the molecular mechanisms underlying the observed substrate switch in pressure overload hypertrophy. These studies have revealed the importance of peroxisome proliferator-activated receptor gamma co-activator (PGC-1α) and peroxisome proliferator-activated receptor (PPAR) in mitochondrial dysfunction. The expression of the PGC-1 gene, as well as nuclear respiratory factor 2 and mitochondrial transcription factor A, which act downstream from PGC-1 were shown to be downregulated in rats with pressure overload due to aortic banding [13]. In a mouse model of transverse aortic constriction pressure overload hypertrophy the mean PPARα mRNA was 39% lower in the hypertrophied animals as compared to controls. Several of PPARα downstream target genes involved in fatty acid oxidation (FAO) were also downregulated [14]. The temporal pattern of the gene expression was characterized in a rat model of progressive LVH and HF [SHHF/Mcc-*facp* (SHHF) rat]. FAO enzyme mRNA levels were coordinately downregulated (>70%) during both the LVH and HF stages in the SHHF rats compared with controls. In contrast, the activity and steady-state levels of medium-chain acyl-CoA dehydrogenase, which catalyzes a rate-limiting step in FAO, were not significantly reduced until the HF stage, indicating additional control at the translational or posttranslational levels in the hypertrophied but nonfailing ventricle [15]. In a rat model of pressure overload hypertrophy, concentrations of free ADP, free AMP, inorganic phosphate (P_i_), and fructose-2,6-bisphosphate (F-2,6-P_2_), all activators of the rate-limiting enzyme phosphofructokinase (PFK), were increased (up to 10-fold) [16]. This activation of PFK alongwith increased glucose uptake contributes to increased glycolysis in this model of pressure overload hypertrophy. These results can be explained by increased intracellular free [ADP] and [AMP], due to decreased energy reserve in LVH, activating the AMP-activated protein kinase cascade. This, in turn, results in enhanced synthesis of F-2,6-P_2_ and increased sarcolemma localization of glucose transporters, leading to coordinated increases in glucose transport and activation of PFK.

## VOLUME OVERLOAD HYPRTROPHY

### Animal Model

Volume overload leads to eccentric hypertrophy as a result of increase in cardiomyocyte length. Left ventricular volume overload can be of two types: low-pressure or high pressure [17]. Low pressure volume overload is the most pure form of volume overload hypertrophy in which the increased volume is pumped into a low pressure chamber. This is seen in cases of mitral regurgitation or ventricular septal defect with normal pulmonary artery pressures. High pressure volume overload occurs when the increased volume is pumped into a high pressure chamber such as aorta. This results in increased pulse pressure and this increase in systolic blood pressure leads to some pressure overload hypertrophy also. This is seen in cases of aortic regurgitation. There have been several animal models of pure volume overloaded LV hypertrophy. In a dog model, chordae tendineae were selectively ruptured by a forceps placed retrograde through the aorta across the aortic valve to the mitral valve apparatus [18]. This resulted in a 50 to 100% increase in LV end diastolic volume within 3 months [19, 20]. These animals developed 30% eccentric hypertrophy and thus there was a decrease in the mass to volume ratio as seen in humans [19, 20]. Left ventricular dysfunction also occurred after 3 months due to 35% loss of the contractile elements [21]. An open chest open heart model of mitral regurgitation has also been created by cutting the chordae tendineae [22]. 

### High Energy Phosphate Levels

Our group has studied the myocardial energetics in a canine model of volume overload LV hypertrophy by tearing the chordae tendineae and causing severe mitral regurgitation (MR) [23]. This resulted in a 30% increase in LV hypertrophy as compared to controls but the LV volume increased by 65% after approximately 1 year. These eccentrically hypertrophied volume overloaded hearts had significantly lower PCr/ATP ratios. Myocardial blood flow and coronary reserve were normal in MR hearts. Moreover hyperperfusion did not correct the abnormal energetics. Interestingly, unlike pressure overload hypertrophy, stresses imposed by exercise, pacing and dobutamine were well tolerated in chronic MR and did not cause further abnormalities in HEP metabolism. 

### Substrate Utilization

Substrate metabolism has also been studied in volume overload LV hypertrophy. In a rabbit model of volume overload created by carotid-jugular shunt formation, eccentric hypertrophy developed as seen by an increase in LV end-diastolic dimension without an increase in anterior or posterior wall thickness [24]. The myocardial uptake of the fatty acid analog ^125^I-iodophenyl 9-methylpentadecanoic acid was increased in the volume-loaded animals, suggestive of a greater fatty acid uptake and oxidation [24]. In contrast to pressure overload hypertrophy, in the early stages of cardiac failure in this model of volume overload eccentric hypertrophy, there was no downregulation of the fatty acid oxidation pathway, but rather evidence for enhanced fatty acid oxidation.

## POST-INFARCTION HYPERTROPHY

### Animal Model

Post-myocardial infarction hypertrophy is a complex phenomenon in which the stretched and dilated infarcted tissue increases left-ventricular volume with a combined volume and pressure load on non-infarcted areas [25]. Our laboratory has created a porcine model of postinfarction remodeling to study myocardial energetics [26]. Proximal circumflex artery was occluded in young pigs and 12 went on to develop left ventricular remodeling (LVR) after about 50 days while 6 developed LVR and CHF within 20 days. The mean scar surface area expressed as a ratio of the total LV surface area was 0.16±0.02 and 0.31±0.03 in the LVR group and LVR+CHF group respectively. This resulted in ventricular hypertrophy in the LVR group as compared to control animals while the LVR+CHF group had the most hypertrophy (LVW/BW was 3.03±0.12* vs. *3.65±0.25* vs. *4.99±0.42). It caused a decrease in ejection fraction to about 35% and 27% in the LVR and LVR+CHF group respectively. LV end-systolic volume increased significantly in both groups of animals with remodeling and was significantly higher in LVR+CHF hearts than in LVR hearts. Both LV diastolic wall stresses and mean LV systolic wall stresses were significantly increased in hearts with LVR and were further increased in LVR+CHF hearts.

### High Energy Phosphate Levels

The bioenergetic abnormalities in our post-infarction LV hypertrophy model were characterized by a lower ATP, CP, creatine and PCr/ATP ratio in the LVR hearts and the LVR+CHF hearts had an even lower PCr/ATP ratio. The myocardial calculated free ADP levels were significantly increased only in CHF hearts. The increase in myocardial free ADP is associated with reductions in mitochondrial F0F1-ATPase protein expression [27]. These HEP levels did not improve with increasing blood flow with adenosine although the subendocardial flow reserve is impaired in CHF animals. There is also a loss of total adenine nucleotide pool in hearts with postinfarction remodeling as shown by accumulation of interstitial purine metabolites found in a rat model of postinfarction remodeling [28]. 

### Myocardial Oxygenation

It is known that the decrease of PCr/ATP in LVR animal during dobutamine infusion is not the result of insufficient myocardial oxygen availability [29]. Furthermore, in CHF hearts, the low basal PCr/ATP and the attenuated response to dobutamine occurred in the absence of myocardial hypoxia, indicating that the HEP and contractile abnormalities were not the result of insufficient oxygen availability [29]. 

### Myocardial Creatine Kinase Kinetics and Isoform Expression

It has been shown that the CK-MM and CK-mitochondrial protein levels are decreased in animals with pos-infarction hypertrophy while the CK-MB levels are increased [30]. However, the total CK activity did not change significantly in the animals with LVH [30]. These changes may partially contribute to the altered HEP metabolism in hearts with postinfarction remodeling. Subsequently, it has been shown that the CK flux rates are decreased in hearts with postinfarction remodeling but this change does not seem to limit their response to dobutamine [31]. However, the animals that develop CHF have further decreased high energy phosphates and CK flux rate and these changes may contribute to their decreased response to dobutamine [31]. 

### Substrate Utilization

Substrate utilization in post-infarction LV hypertrophy also exhibits shifts in fatty acid and glucose metabolism. In a post-infarction rat model, glucose oxidation was increased by 84% while there was no change in palmitate oxidation 8 weeks after MI [32]. The pattern of gene expression regulating substrate utilization could change as LV remodeling progresses to heart failure, so the same group followed these animals for 20 weeks [33]. At 8 weeks, when left ventricular hypertrophy was present without signs of heart failure, myocardial mRNA expression of glucose transporters (GLUT-1 and GLUT-4) was not altered, whereas mRNA expression of medium-chain acyl-CoA dehydrogenase (MCAD) was significantly reduced in the peri-infarct region. In hearts exhibiting heart failure 20 weeks after infarct-induction there was a change of both mRNA and protein content of GLUT-1 and MCAD [33].

### Infarct Borderzone Energetic Abnormalities

Post-infarction hypertrophy differs significantly from other forms of hypertrophy because of the presence of a discrete scar region. The alterations in myocardial HEP and mtATPase have been shown to be substantially more severe in the peri-infarct region than in the remote zone of hearts with compensated postinfarction LV remodeling [34]. These abnormalities mirror the contractile dysfunction of the border zone myocardium and may play a role in the eventual transition to heart failure. The bioenergetic and contractile abnormalities of the BZ, which are triggered by elevated regional wall stress, may extend laterally and eventually involve the entire LV, thereby leading to global LV dysfunction and the development of CHF [35].

## HUMAN CLINICAL STUDIES

Clinical studies of cardiac energy metabolism (summarized in Table **2**) have shown similar results to previously obtained from animal studies. 

The PCr/ATP ratio is decreased in patients with pressure induced LVH [36, 37]. In a study of patients with diastolic dysfunction secondary to hypertensive heart disease, patients showed higher LV mass (98±28 g/m^2^) than healthy control subjects (73±13 g/m^2^, *P*<0.01). LV filling was impaired in patients, reflected by a decreased peak rate of wall thinning (PRWThn), E/A ratio, early peak filling rate, and early deceleration peak (all *P*<0.05), whereas systolic function was still normal. The myocardial PCr/ATP ratio determined in patients at rest (1.20±0.18) and during stress (0.95±0.25) was lower than corresponding values obtained from healthy control subjects at rest (1.39±0.17, *P*<0.05) and during stress (1.16±0.18, *P*<0.05). The PCr/ATP ratio correlated significantly with PRWThn (*r*=-0.55, *P*<0.01), early deceleration peak (*r*=-0.56, *P*<0.01), and with the rate-pressure product (*r*=-0.53, *P*<0.001). Creatine kinase kinetics is also affected in patients with pressure induced LVH. In hypertrophic human hearts in the absence of CHF, ATP flux through CK is reduced by 30% in LVH patients, and this reduction can be accounted for by a proportionate loss of substrate (PCr) [38]. Patients with a similar degree of LVH but who additionally had CHF exhibited no greater reductions in PCr and ATP than those without CHF. However, the CK pseudo first-order rate constant was markedly depressed in these CHF patients compared with that of normal subjects and of LVH patients without CHF. Consequently, myocardial CK flux in LVH patients with CHF declined to approximately one third that of normal subjects [38]. These data provide direct evidence that a defect in the kinetics of ATP turnover through CK and not the pool sizes per se may distinguish failing and nonfailing human hypertrophic myocardium. 

PCr/ATP is also lower in humans with volume overload hypertrophy such as chronic MR and the severity of abnormal myocardial energetics is linked to disease severity as measured by dimensional indexes of left ventricular dilatation and hypertrophy [39]. In patients with dilated cardiomyopathy, PCr/ATP ratio was lower and seemed to correlate with worsening heart failure as assessed by NYHA class [40]. It has also been suggested that PCr/ATP ratios are a predictor of both total and cardiovascular mortality in patients with dilated cardiomyopathy [41]. In another study, patients with DCM were also shown to have a reduction in absolute concentrations of PCr by 51% and ATP by 35% [42]. CK activity is also lower in patients with DCM undergoing transplantation and this was associated with decreased expression of CK-MM and increased expression of CK-MB [43]. 

There have been some myocardial energetic studies in ischemic heart disease patients. In patients with ischemic cardiomyopathy, PCr/ATP ratio was significantly lower as compared to controls [44]. Cardiac energy metabolism is normal at rest in patients with chronic myocardial infarction without heart failure [40]. However, this ratio significantly decreases during exercise [45]. In another study, patients with ischemic heart disease had significantly lower PCr content if they had either a reversible or fixed defect on exercise ^201^Tl scintigraphy, but ATP content was decreased significantly only in subjects with fixed thallium defects [46]. 

The data on substrate metabolism in patients with LV hypertrophy is scant, while in patients with congestive heart failure it is conflicting. In a study of NYHA class II and III patients, fatty acid uptake was increased while glucose uptake was decreased [47]. This was associated with a 50% increase in lipid oxidation as measured by transmyocardial respiratory quotient and a 60% decrease in carbohydrate oxidation. It was also demonstrated in a PET study that patients with NYHA class III heart failure (majority ischemic) had greater myocardial uptake of a radiolabeled fatty acid analog and less uptake of radiolabeled deoxyglucose [48]. On the other hand, in dilated cardiomyopathy patients, fatty acid utilization was decreased and myocardial glucose metabolism was increased [49]. Overall, it is believed that in the early stages of heart failure there is a normal (or slightly elevated) rate of fatty acid oxidation, with a dramatic downregulation of fatty acid oxidation in advanced or end-stage heart failure [50].

## CONCLUSION

Left ventricular hypertrophy is characterized by derangements in cardiac energy metabolism including lower PCr/ATP, abnormal creatine kinase kinetics and deranged substrate utilization. The PCr/ATP is significantly decreased in hearts with LVH and the severity of this abnormality is proportional to the degree of cardiac hypertrophy and accompanying LV dysfunction. These myocardial energetic abnormalities are also not due to a persistent myocardial ischemic state. There is a profound heterogeneity in the myocardial energy metabolism in hearts with postinfarction LV remodeling with the most severe abnormality occurring in the inner layers of the periscar border zone (BZ). Increased wall stress and overstretching of myocytes could contribute to the observed decrease in energetic efficiency in the BZ. CHF probably develops as this energetically inefficient BZ expands over time and eventually involves the entire heart. 

Preserving myocardial energetic efficiency through metabolic therapies is a promising therapy for heart failure in the future. To achieve this goal, further molecular studies are required to understand the mechanisms underlying the myocardial energetic abnormalities. Furthermore, development of effective and time feasible methods of non-invasive NMR spectroscopy along with magnetic resonance imaging (MRI) will allow us to study temporal changes in myocardial energetics in patients with LVH and CHF and correlate these with structural changes in the hearts. Finally, new drugs need to be developed and tested that target energy metabolism pathways with the ultimate goal of improving survival in patient with LVH and CHF.

## Figures and Tables

**Table 1:** Myocardial Energetics in Different Animals Models of Left Ventricular Hypertrophy

	Pressure Overload Hypertrophy	Volume Overload Hypertrophy	Post-infarction Hypertrophy
PCr/ATP under baseline conditions	Decreased in severe LVH but normal in moderate LVH.	Decreased	Decreased
PCr/ATP under increased workload	Decreased in both moderate and severe LVH.	No further decrease in PCr/ATP from baseline.	Decreased
Regional heterogeneity in PCr/ATP	Worse in subendocardium.	-	Worse in the borderzone subendocardium
Are changes in PCr/ATP due to ischemia?	No	No	No
CK forward rate constant	No change	-	No change
CK flux rate	Decreased	-	Decreased
Total CK activity	Normal	-	Normal
CK-MM isoform	Decreased	-	Decreased
CK-mitochondrial isoform	Decreased	-	Decreased
CK-MB isoform	Increased	-	Increased
Fatty acid oxidation	Decreased	Increased	No change
Glycolysis	Increased	-	Increased
Glucose oxidation	Increased probably through anaplerotic pathways	-	Increased

PCr, Phosphocreatine; ATP, Adenosine Triphosphate; CK, Creatine Kinase.

**Table 2:** Myocardial Energetics in Humans with Left Ventricular Hypertrophy

	Pressure Overload Hypertrophy	Volume Overload Hypertrophy	Post-infarction Hypertrophy
PCr/ATP under baseline conditions	Decreased	Decreased	Decreased
PCr/ATP under increased workload	Decreased	-	Decreased
CK forward rate constant	No change with LVH but decreased with LVH and CHF.	-	-
CK flux rate	Decreased	-	-
CK-MM isoform	-	Decreased	-
CK-MB isoform	-	Increased	-
Substrate Utilization		Conflicting data across groups but it seems that in early stages of heart failure there is normal or slightly elevated rate of fatty acid oxidation with a dramatic downregulation in advanced heart failure.	

PCr, Phosphocreatine; ATP, Adenosine Triphosphate; CK, Creatine Kinase.
